# RETRACTED: Mannino et al. Beta-Caryophyllene, a Plant-Derived CB2 Receptor Agonist, Protects SH-SY5Y Cells from Cadmium-Induced Toxicity. *Int. J. Mol. Sci.* 2023, *24*, 15487

**DOI:** 10.3390/ijms26051805

**Published:** 2025-02-20

**Authors:** Federica Mannino, Giovanni Pallio, Chiara Imbesi, Alessandro Scarfone, Domenico Puzzolo, Antonio Micali, José Freni, Francesco Squadrito, Alessandra Bitto, Letteria Minutoli, Natasha Irrera

**Affiliations:** 1 Department of Clinical and Experimental Medicine, University of Messina, 98125 Messina, Italy; fmannino@unime.it (F.M.); chimbesi@unime.it (C.I.); alessandro.scarfone@unime.it (A.S.); fsquadrito@unime.it (F.S.); abitto@unime.it (A.B.); nirrera@unime.it (N.I.); 2 Department of Biomedical and Dental Sciences and Morphological and Functional Imaging, University of Messina, 98125 Messina, Italy; gpallio@unime.it (G.P.); puzzolo@unime.it (D.P.); jose.freni@unime.it (J.F.); 3 Department of Adult and Childhood Pathology “Gaetano Barresi”, University of Messina, 98125 Messina, Italy; amicali@unime.it

The journal retracts the article titled “Beta-Caryophyllene, a Plant-Derived CB2 Receptor Agonist, Protects SH-SY5Y Cells from Cadmium-Induced Toxicity” [1], cited above.

Following publication, concerns were brought to the attention of the Editorial Office regarding the duplication of images within this article [1].

Adhering to our standard procedure, the Editorial Office and Editorial Board conducted an investigation that confirmed the duplication of Figure 5B,C. While the authors cooperated with the investigation, they were unable to satisfactorily explain the duplication or provide suitable raw material for the Editorial Board’s evaluation. Consequently, the Editorial Board has lost confidence in the reliability of the findings and decided to retract this publication [1], as per MDPI’s retraction policy (https://www.mdpi.com/ethics#_bookmark30).

This retraction was approved by the Editor-in-Chief of the *Int. J. Mol. Sci.* journal. The authors did not agree to this retraction.
